# CGMS and Glycemic Variability, Relevance in Clinical Research to Evaluate Interventions in T2D, a Literature Review

**DOI:** 10.3389/fendo.2021.666008

**Published:** 2021-09-09

**Authors:** Anne-Esther Breyton, Stéphanie Lambert-Porcheron, Martine Laville, Sophie Vinoy, Julie-Anne Nazare

**Affiliations:** ^1^Centre de Recherche en Nutrition Humaine Rhône-Alpes, Univ-Lyon, CarMeN Laboratory, INSERM, INRA, INSA Lyon, Université Claude Bernard Lyon 1, Hospices Civils de Lyon, F-CRIN/FORCE Network, Pierre Bénite, France; ^2^Nutrition Research, Mondelez International, Saclay, France; ^3^Department of Endocrinology Diabetes and Nutrition, Centre Hospitalier Lyon Sud, Hospices Civils de Lyon, Pierre Bénite, France

**Keywords:** glycemic variability, type 2 diabetes, continuous glucose monitoring system, clinical research, interventions

## Abstract

Glycemic variability (GV) appears today as an integral component of glucose homeostasis for the management of type 2 diabetes (T2D). This review aims at investigating the use and relevance of GV parameters in interventional and observational studies for glucose control management in T2D. It will first focus on the relationships between GV parameters measured by continuous glucose monitoring system (CGMS) and glycemic control and T2D-associated complications markers. The second part will be dedicated to the analysis of GV parameters from CGMS as outcomes in interventional studies (pharmacological, nutritional, physical activity) aimed at improving glycemic control in patients with T2D. From 243 articles first identified, 63 articles were included (27 for the first part and 38 for the second part). For both analyses, the majority of the identified studies were pharmacological. Lifestyle studies (including nutritional and physical activity-based studies, N-AP) were poorly represented. Concerning the relationships of GV parameters with those for glycemic control and T2D related-complications, the standard deviation (SD), the coefficient of variation (CV), the mean blood glucose (MBG), and the mean amplitude of the glycemic excursions (MAGEs) were the most studied, showing strong relationships, in particular with HbA1c. Regarding the use and relevance of GV as an outcome in interventional studies, in pharmacological ones, SD, MAGE, MBG, and time in range (TIR) were the GV parameters used as main criteria in most studies, showing significant improvement after intervention, in parallel or not with glycemic control parameters’ (HbA1c, FBG, and PPBG) improvement. In N-AP studies, the same results were observed for SD, MAGE, and TIR. Despite the small number of N-AP studies addressing both GV and glycemic control parameters compared to pharmacological ones, N-AP studies have shown promising results on GV parameters and would require more in-depth work. Evaluating CGMS-GV parameters as outcomes in interventional studies may provide a more integrative dimension of glucose control than the standard postprandial follow-up. GV appears to be a key component of T2D dysglycemia, and some parameters such as MAGE, SD, or TIR could be used routinely in addition to classical markers of glycemic control such as HbA1c, fasting, or postprandial glycemia.

## Introduction

In 2019, the International Diabetes Federation (IDF) estimates that one in 11 adults (20–79 years) has diabetes, which represents 463 million people worldwide. By 2030, this will rise to 578 million, and by 2045, this could reach 700 million. Type 2 diabetes (T2D) is the most common type of diabetes, accounting for around 90% of all diabetes worldwide, increasing each year in most countries with around 374 million people at increased risk of developing T2D (1, 2). Diabetes complications already caused 4.2 million deaths in 2019 and are estimated to be associated with 11.3% of global deaths from all causes among people in the same age group. T2D leads to many complications, mainly due to complex and interconnected mechanisms combining hyperglycemia, insulin-resistance, low-grade inflammation, and accelerated atherogenesis. Cardiovascular disease, such as coronaropathy, stroke or, heart failure is often associated with T2D, which makes it an independent cardiovascular risk factor. T2D hyperglycemia could also affect kidneys and alters their function leading to microalbuminuria and a diabetic nephropathy (3, 4). Retinopathy is another serious complication in T2D people which have a higher risk of blindness than people without diabetes (4). T2D is considered as a complex and progressive disease, characterized by glycemic disorders including both sustained chronic hyperglycemia and acute glucose fluctuations (5, 6). Beyond fasting hyperglycemia, postprandial hyperglycemia contributes to total glycemic exposure and appears to be a great predictor of cardiovascular risk in T2D people (7). Caring about postprandial state in T2D people profile seems quite relevant considering the large time spent in this postprandial state within a day. However, controlling postprandial glucose level appears as an important strategy to prevent cardiovascular complications associated with diabetes (7). Ever since the completion of two randomized, interventional studies, the Diabetes Control and Complications Trial in type 1 diabetes (T1D) and the United Kingdom Prospective Diabetes Study in T2D, HbA1c has become the basis for understanding the relationship of glycemic control with micro- and macrovascular complications (8, 9). Both studies showed that a reduction in HbA1C was associated with a reduction in these complications. HbA1c, the diabetes gold standard, is of course an integrator of both fasting and postprandial glycemic disorders (5); however this parameter will not dissociate fasting than postprandial state and will reflect more chronic dysglycemia than acute ones. Glycemic variability (GV) is now recognized as an integral component of glucose homeostasis (10, 11), including various measurements such as the percent of time within the target range for glucose or the frequency/duration/severity of hyperglycemia and hypoglycemia. Although considering GV as an independent risk factor for diabetes complications is not definitively established for T2D people, GV allows the assessment of the presence of excess glycemic excursions, and therefore the risk of hyper or hypoglycemia (10). As a consequence, simultaneously taking into account HbA1c, fasting and postprandial glucose and GV in T2D management could be part of an interesting strategy aiming at improving glycemic control. This review will firstly focus on the relationships of GV parameters with T2D diagnosis and glucose control parameters and T2D related complications. The second objective will be to investigate the use of GV as a relevant outcome in interventional studies (pharmacological, nutritional, physical activity) aiming at improving glycemic control in T2D patients. For this review, we specifically choose to evaluate GV by CGM for several reasons: it is a novel methodology providing the most precise and representative information related to glycemic response, thanks to a great number of glycemic data, and calculated variables were compared to other routine glycemia follow-up methodologies.

## Glycemic Variability: Concept, Definition And Use

The chronic glycemic disorders have been well documented, and there is now cogent evidence for the deleterious effects of such chronic hyperglycemia, generating oxidative stress among others (5). The role of acute glucose fluctuations, which can be defined as GV, including fasting and postprandial state, into diabetes complications and management has been less documented (5). Continuous glucose monitoring is an emerging technique of the last decade which appears to be very useful for T2D management and care. According to the American Diabetes Association (ADA) recommendations, the use of CGMS is advocating for children and youths with T1D but, so far, no recommendations have been settled for type 2 diabetes people without insulin therapy (12). The most common technique of continuous glucose measurement relies on a subcutaneous system, measuring an electric signal in the interstitial liquid, providing a measure of glycemia every 5 min, meaning 288 reading per day of CGMS holding period. Whether for a personal (real time version) or a professional (masked version) use, the daily glycemic profile obtained can be really helpful in detecting specific periods or time of nocturnal or asymptomatic hypo and hyperglycemia in order to adjust patient treatment or to collect data about such episodes in some clinical studies’ point of view (13, 14). Those devices allow a global overview of glycemic fluctuations over 24 h, much more precise than the one supplied by some self-monitoring blood glucose (SMBG) device (13).

Thanks to these devices, various and numerous metrics assessing GV are affordable, each one having its own relevance concerning GV. **Table 1** summarizes the available parameters used, their description, and computation (16–24).

**Table 1:** Glycemic variability parameters. * (15).

Parameter	Formula	Description
Standard Deviation (mg/dL)	$\text{SD}=\sqrt{\frac{\Sigma_{t=t_{1}}^{t_{n}}{\left(BG_{t_{i}}-\bar{\text{BG}}\right)}^{2}}{n-1}}$	where n = number of glycemic values and BG = glycemic value
Coefficient of Variation (%)	$\text{CV}=\left(\frac{SD}{\bar{BG}}\right)\times 100$	
Mean Amplitude of Glucose Excursions (mg/dL)	$\text{MAGE}=\Sigma \frac{\lambda }{x},if\;\lambda >\nu$	where λ = absolute value difference between sequential glucose peaks and nadirs; x = number of valid observations and υ = 1 SD of mean glucose
Mean Of Daily Differences	$\text{MODD}=\frac{\Sigma_{t=t_{1}}^{t_{k}}\left|BG_{t}-BG_{t-1440}\right|}{\text{k}}$	where k = number of observation where there is an observation at the same time 24h (1440 min) ago
Mean Indices of Meal Excursions (MIME)		
Glycemic Delta (mg/dL)	$\Delta \text{G}=G_{T_{\max}}-G_{T_{0}}$	where G_Tmax_ = glycemic value at T_max_ and G_T0_ = glycemic value at T_0_
Time Delta (min)	ΔT = T_max_ – T_0_	where T_max_ = time of the postprandial glycemic peak and T_0_ = time of the meal’s beginning
Basal Return (%)	$\text{RB}=\frac{\Delta G\times 100}{G_{T_{\max}}-G_{T_{\max+1h}}}$	where G_Tmax+1h_ = glycemic value at T_max+1h_
Interquartile 50 (mg/dL)	IQR50 = Q_3_ – Q_1_	where Q_1_ is the first quartile and Q_3_ is the third quartile
Time In Range (%)		TIR represents the time spent by each subject in a specific glycemic range; 5 glycemic ranges were defined: <70 mg/dL; [70-140 mg/dL[; [140-180 mg/dL[; [180-250 mg/dL]; and >250 mg/dL
Low Blood Glucose Index	$\text{LBGI}=\frac{\Sigma_{i=1}^{n}r_{l}(BG_{i})}{n}$	where r_l_(BG_i_) = 22.77 x f(BG_i_)^2^, if f(BG_i_) < 0 and 0 otherwise; and f(BG_i_) = (ln(BG_i_)^1.084^ – 5.381)
High Blood Glucose Index	$\text{HBGI}=\frac{\Sigma_{i=1}^{n}r_{h}(BG_{i})}{n}$	where r_h_(BG_i_) = 22.77 x f(BG_i_)^2^, if f(BG_i_) > 0 and 0 otherwise; and f(BG_i_) = (ln(BG_i_)^1.084^ – 5.381)
Average of Daily Risk Ratio	$\text{ADRR}=\frac{1}{\text{M}}\sum_{j=1}^{M}\left(LR^{j}+HR^{j}\right)$	where M = days of measurement and where LR^j^ = max(r_l_(BG_1_), …, r_l_(BG_k_)) and HR^j^ = max(r_h_(BG_1_), …, r_h_(BG_k_)) are the maximum hypo and hyperglycemia risk values for day j, j = 1, 2, …, M.
Minimum of glycemia (mg/dL)	Min Gly = Min(*BG*_1_, *BG*_2_, …, *BG_n_*)	where n = number of glycemic values
Maximum of glycemia (mg/dL)	Max Gly = Max(BG_1_, BG_2_, …, BG_n_)	where n = number of glycemic values
Continuous Overall Net Glycemic Action	$\text{CONGA}(\text{n})=\sqrt{\frac{\Sigma_{t=t_{1}}^{t_{k}}{\left(D_{t}-\bar{D}\right)}^{2}}{k-1}}$	where D_t_ = BG_t_ – BG_t-m_ ; k = number of observations where there is an observation n x 60 min ago and m = n x 60
J-index	J = 0.001 (MBG + SD)^2^ (1) J = 0.324 (MBG + SD)^2^ (2)	where MBG = mean blood glucose and SD = standard deviation (1) for glucose measured in mg/dL (2) for glucose measured in mmol/L
Largest Amplitude of Glycemic Excursion	LAGE = G_max_ – G_min_	where G_max_ = maximum glucose measured and G_min_ = minimum glucose measured
Index of Glycemic Control	*IGC* = *LGBI* + HGBI	
Fractal Dimension		The calculation is based on the changes of glucose values between subsequent measurements using the Higuchi algorithm*

In a recent report, three randomized trials, including more than 380 participants and measuring mean glucose with CGMS *versus* HbA1c measured in central laboratories, showed that HbA1c may underestimate or overestimate mean glucose. Henceforth, a patient’s CGMS profile has considerable potential for optimizing his or her glycemic management (25). Accordingly, this is about offering an additional support to HbA1c metrics to assess and anticipate the risk of an individual developing diabetes associated complications, and it is in this context that GV concept was highlighted. According to Hirsch (26), this GV could be defined as the degree to which a patient’s blood glucose level fluctuates between high (peaks) and low (nadir) levels. Due to the recent interest for this new concept, the best and most precise way to assess GV is still debated, and a large number of parameters are available, each one trying to cover a specific field of the GV. Although there is a consensus that HbA1c remains the current gold standard for the primary clinical target, there is no global agreement concerning the GV metrics able to provide additional clinical data or to become additional targets beyond HbA1c (26).

## Glycemic Variability And T2d: Evaluation Of Relationship And Assessment Of Outcomes In Interventional Studies

### Material And Methods

This work was done following two distinct objectives: the evaluation of the relationships between GV and T2D markers focusing on using CGMS-derived GV metrics in the assessment of glycemic control (Part 1) and the assessment of GV as a relevant outcome in interventional studies for improving T2D management (Part 2). For this, we included studies that collected both 1) GV parameters, obtained thanks to any type of CGMS (masked version), and 2) T2D diagnosis or metabolic disorder-related markers. Only studies focusing on T2D people over 18 years old who underwent a period with any type of glucose monitoring system were included. There was no restriction on medication or in the selection of our studies. Studies were not included if they involved animals, children, or adolescents and if they did not involve associations including GV parameters. Studies focusing on real-time CGMS were not included. Only articles written in English and French languages were included. Studies focusing on specific age group, specific sex or gender, targeting a specific diabetes complication or a specific medication with a mention of each specificity were included.

For both analyses (1. relationship and 2. outcomes), electronic databases, such as PubMed or Scopus were used to search for relevant studies from articles published in English or in French after 1990. The search was updated with recent papers until 2020. Combinations of the following key words were used: human, glycemic variability, correlation, association, type 2 diabetes, continuous glucose monitoring, diet, intervention. Three reviewers (A-EB, J-AN, and SV) independently screened citations and abstracts to identify articles potentially meeting the inclusion criteria for each analysis, indicating for which one the article fitted (relationship analysis or outcome analysis). For those articles, full text versions were retrieved and independently screened by two reviewers (AE-B and J-AN or A-EB and SV) to determine if they met or not the inclusion criteria for each analysis. Any discord concerning the inclusion criteria was resolved through a discussion with a third reviewer (J-AN or SV). Data extraction of relevant study information for selected articles was performed by two independent reviewers (A-EB and J-AN or A-EB and SV or A-EB and S-LP) and discords were resolved by discussion. For each analysis, a data extraction form was used to collect information on the article (authors, title, source, year), the study population and baseline measurements, the study design, the study duration, the type of glucose monitoring used, the outcome measures, and main findings.

#### Part 1: Association Between GV Parameters and T2D Markers

The main goal was to assess relationship between GV parameters [standard deviation (SD), coefficient of variation (CV), mean amplitude of glucose excursion (MAGE), mean of daily differences (MODD), mean indices of meal excursion (MIME), interquartile 50 (IQR50), time in range (TIR), low blood glucose index (LBGI), high blood glucose index (HBGI), average of daily risk ratio (ADRR), minimum of glycemia (Min Gly), maximum of glycemia (Max Gly), continuous overall net glycemic action (CONGA), largest amplitude of glucose excursion (LAGE), mean blood glucose (MBG), J-index, fractal dimension (FD), index of glycemic control (IGC)] and glycemic control [HbA1c, fasting blood glucose (FBG), postprandial blood glucose (PPBG), C-peptide, adiponectin] and T2D-related metabolic disorder markers [glucose overall exposure with fructosamine (FA), glycated albumin (GA) and 1,5-anhydroglucitol (1,5-AG); oxidative stress with 15 F2t isoprostane (15-isoP F2t), 8-iso-prostaglandin F2*α* (8-Iso-PGF2*α*), derivatives of reactive oxygen metabolites (d-ROMs), thiobarbituric acid reactive substances (TBARS) and glutathione (GSH); microvascular complications with urinary albumin/creatinine ratio (UACR), diabetic retinopathy (DR), diabetic peripheral neuropathy (DPN) and medial plantar compound nerve action potential (CNAP); cardiovascular risk with baroreflex sensitivity (BRS), flow-mediated dilation (FMD) and left ventricular mass index (LVMI); blood pressure with diastolic blood pressure (DBP) and systolic blood pressure (SBP) and sympathovagal balance with low frequency/high frequency ratio (LF/HF)].

For this work, we assessed different types of relationships including correlations, linear regressions, logistic regressions, and mixed models analyses. The table built for this part summarizes all relationships reported in the selected studies with information regarding significance level, relationship direction, and type of statistical analysis conducted. Priority was given to regression analyses over correlation analyses.

#### Part 2: GV as an Outcome in Interventional Studies for Improving T2D Management

In this analysis, only interventional studies were included. Main assessments were changes in GV parameters (SD, CV, MAGE, MODD, MIME, IQR50, TIR, LBGI, HBGI, ADRR, Min Gly, Max Gly, CONGA, LAGE, MBG, J-index, and FD) after an intervention (pharmacological, nutritional, or based on physical activity). Other assessments were changes in HbA1c, FBG, and PPBG.

For this work, we built a timeline representation based on the number of studies evaluating changes for each GV parameters and glycemic control (HbA1c, FBG, PPBG) at different time points. We indicated the level of significance for each change, the type of study (pharmacological, nutritional or based on physical activity), and the duration of the study.

### Results

#### Part 1: Relationships Between GV Parameters and T2D Markers

**Figure 1:**
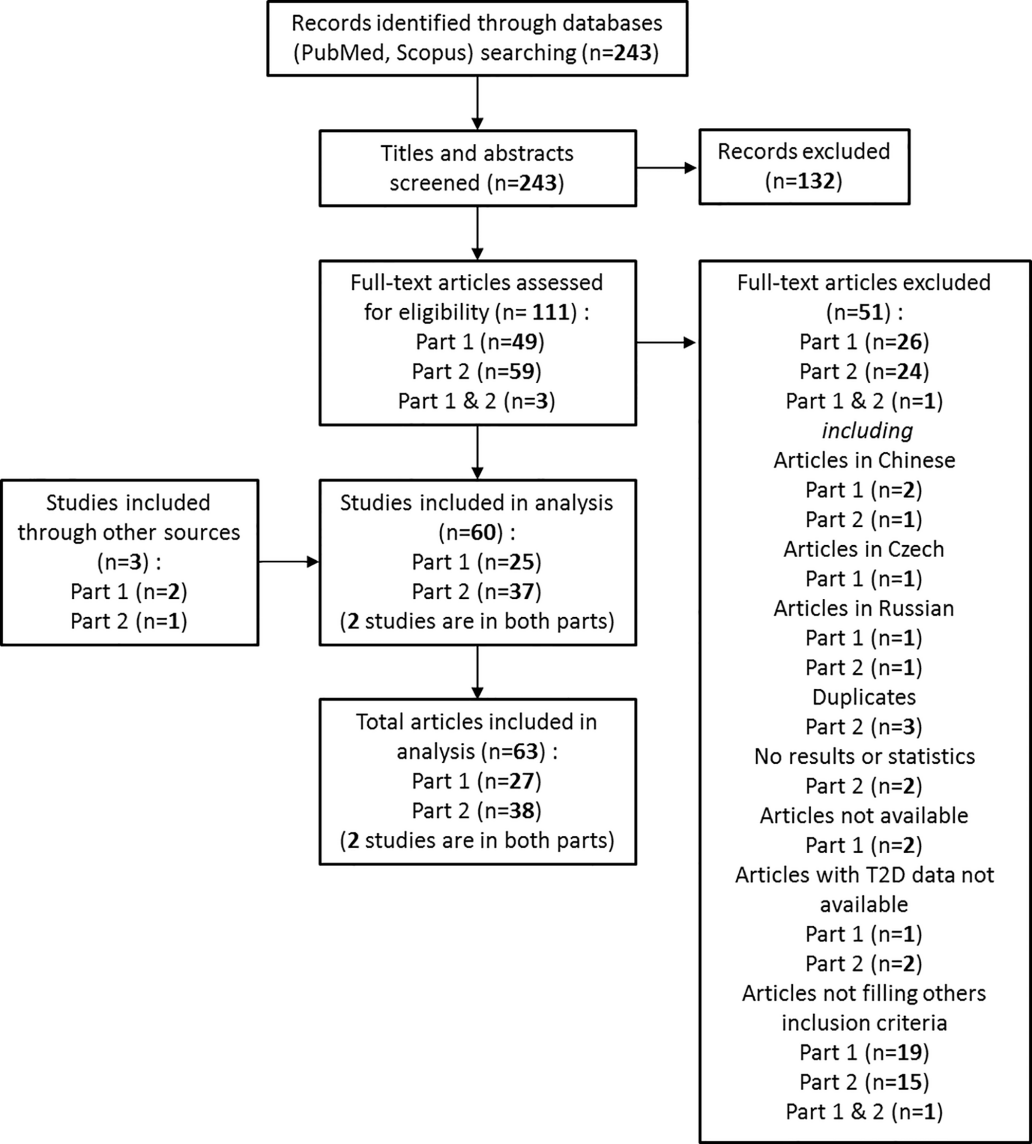
Flow chart of the review.

Article screening is presented in the flowchart (**Figure 1**). The initial search identified a total of 243 records. After scanning titles and abstracts, 52 were accepted for further screening, and full-text of these studies was reviewed. Of these, 25 studies were identified as meeting the inclusion criteria. In addition, two articles were identified through other sources during the process. In total, 27 articles were included. Common reasons for exclusion were: inclusion criteria not fulfilled, languages other than English or French, and full-text unavailability.

The characteristics of included studies are presented in supplemental data (**Table S1**). Among the 27 studies, three were based on interventional studies (two pharmacological intervention studies and one physical activity intervention study) and 24 were cross-sectional studies. Eighteen studies were done on T2D using insulin as basal treatment (among others), and five were conducted on T2D people without insulin treatment.

Concerning the GV markers and their relationships with T2D and complication related markers, 16 studies evaluated SD, 13 evaluated CV, 23 evaluated MAGE, 13 evaluated MBG, seven evaluated MODD, four evaluated MIME ΔG, none evaluated MIME ΔG or MIME BR, one evaluated IQR50, thee evaluated TIR, two evaluated LBGI, two evaluated HGBI, one evaluated ADRR, none evaluated Min gly or Max gly, six evaluated CONGA, four evaluated J-index, one evaluated FD, and one evaluated IGC.

Concerning the T2D and metabolic disorder-related markers and their relationships with GV markers, 16 studies evaluated HbA1c, six evaluated FPG, one evaluated PPBG, five evaluated C-peptide, one evaluated adiponectin, four evaluated glucose overall exposure markers, seven evaluated oxidative stress markers, five evaluated microvascular complication markers, two evaluated cardiovascular risk markers, and two evaluated blood pressure and with sympathovagal balance markers.

##### MAGE

MAGE appears to be the most studied GV parameter in terms of relationships with glycemic control and metabolic disorder-related markers. In our analysis, MAGE was the only GV parameter whose relationships have been evaluated for all the T2D or metabolic disorder type-related markers.

Sixteen relationships between MAGE and HbA1c were studied including 11 correlation analyses (five non-significant and six positive), four linear regression analyses (one non-significant and four positive) and one positive logistic regression analysis. Of the 15 studies presenting tested relationships (one study tested this relationship in two different subgroups) nine were done on more than 50 subjects and the remaining six studies recruited at least 24 subjects. In total, relationships between MAGE and HbA1c were tested on 1,417 T2D patients. All studies were dealing with males and females, with a CGMS hold between 2 and 7 days. For the studies in which information was available, diabetes duration was between 4 and 10 years in four studies and above 10 years for seven studies. In studies adjusting for parameters, such as age or HbA1c, the relationship was maintained after adjustment.

Five correlations between MAGE and FBG or PPBG were studied. Two were non-significant, and three were positively correlated. In total, relationships between MAGE and FBG or PPBG were tested on 499 T2D patients. Four relationships between MAGE and C-peptide were studied including one non-significant correlation analysis and three linear regression analyses (one non-significant and two negative). In total, relationships between MAGE and C-peptide were tested on 628 T2D patients. Seven relationships between MAGE and glucose overall exposure markers were studied including four correlation analyses (one non-significant, two positive, and one negative), one positive linear regression analysis and two mixed model analyses (one non-significant and one positive). In total, relationships between MAGE and glucose overall exposure markers were tested on 259 T2D patients. Seven relationships between MAGE and oxidative stress markers were studied including four correlations analyses (one non-significant, two positive, and one negative) and three linear regression analyses (one non-significant and two positive). In total, relationships between MAGE and oxidative stress markers were tested on 238 T2D patients. Five relationships between MAGE and microvascular complication markers were studied including one negative linear regression analysis and four logistic regression analysis (one non-significant and three positive). In total, relationships between MAGE and microvascular complication markers were tested on 4,109 T2D patients. Three relationships between MAGE and cardiovascular risk markers were studied including two non-significant correlation analyses and one negative linear regression analysis. In total, relationships between MAGE and cardiovascular risk markers were tested on 120 T2D patients. Five correlations between MAGE and BP and sympatho-vagal balance markers were studied, and all were positive. In total, relationships between MAGE and BP and sympatho-vagal balance markers were tested on 86 T2D patients.

##### SD

SD takes the second position in terms of the number of studies assessing SD relationships (16/27 studies). Twelve relationships between SD and HbA1c were studied including five correlation analyses (two non-significant and three positive) and seven linear regression analyses (one non-significant and six positive). In total, relationships between SD and HbA1c were tested on 1,295 T2D patients. Only one positive correlation was tested between SD and FBG on 114 T2D patients; however no relationship was studied with PPBG. Four relationships between SD and C-peptide were studied including one non-significant correlation analysis and three linear regression analyses (one non-significant and two negative). In total, relationships between SD and C-peptide were tested on 621 T2D patients. Seven relationships between SD and glucose overall exposure markers were studied including three correlations analyses (two positive and one negative), two positive linear regression analysis and two non-significant mixed model analyses. In total, relationships between SD and glucose overall exposure markers were tested on 199 T2D patients. Four logistic regression analyses (one non-significant and three positive) between SD and microvascular complications markers were studied on 4,082 T2D patients. We noticed the lack of evaluated relationships between SD and oxidative stress, cardiovascular risk and blood pressure markers.

##### CV

CV takes the third position (equally with MBG) in terms of the number of studies assessing CV relationships (13/27 studies) with almost the same type of relationships as SD and the same characteristics as previously described. Eight relationships between CV and HbA1c were studied including two correlation analyses (one non-significant and one positive) and six linear regression analyses (four non-significant and two positive). In total, relationships between CV and HbA1c were tested on 907 T2D patients. Only one non-significant correlation was tested between CV and FBG on 60 T2D patients; however no relationship was studied with PPBG. Seven relationships between CV and C-peptide were studied including one non-significant correlation analysis and six linear regression analyses (one non-significant and five negative). In total, relationships between CV and C-peptide were tested on 1,125 T2D patients. Six relationships between CV and glucose overall exposure markers were studied including three correlations analyses (two positive and one negative), one non-significant linear regression analysis and two non-significant mixed model analyses. In total, relationships between CV and glucose overall exposure markers were tested on 199 T2D patients. We noticed the lack of evaluated relationships between CV and oxidative stress, microvascular complications, cardiovascular risk, and blood pressure markers.

##### MBG

MBG takes the third position (equally with CV) in terms of studies number assessing MBG relationships (13/27 studies). Nine relationships between MBG and HbA1c were studied including seven correlation analyses (two non-significant and six positive) and two positive linear regression analyses. In total, relationships between MBG and HbA1c were tested on 450 T2D patients. Three correlation analyses (one non-significant and two positive) were tested between MBG and FBG on 130 T2D patients; however no relationship was studied with PPBG. Eight relationships between MBG and glucose overall exposure markers were studied including four correlations analyses (two positive and two negative), two positive linear regression analyses and two positive mixed model analyses. In total, relationships between MBG and glucose overall exposure markers were tested on 259 T2D patients. We noticed the lack of evaluated relationships between MBG and oxidative stress, microvascular complications, cardiovascular risk, and blood pressure markers.

##### MODD

MODD takes the fourth position in terms of the number of studies assessing MODD relationships (7/27 studies). Four relationships between MODD and HbA1c were studied including two correlation analyses (one non-significant and one positive) and two positive linear regression analyses. In total, relationships between MODD and HbA1c were tested on 586 T2D patients. Two positive linear regression analyses were tested between MODD and FBG on 209 T2D patients; however no relationship was studied with PPBG. Three positive linear regression analyses between MODD and C-peptide were studied on 575 T2D patients. Five relationships between MODD and glucose overall exposure markers were studied including four correlations analyses (one non-significant, two positive, and one negative) and one positive linear regression analyses. In total, relationships between MODD and glucose overall exposure markers were tested on 220 T2D patients. Three logistic regression analyses (one non-significant and two positive) between MODD and microvascular complications markers were studied on 1,155 T2D patients. We noticed the lack of evaluated relationships between MODD and oxidative stress, cardiovascular risk, and blood pressure markers.

##### CONGAs and MIME ΔG

CONGA (this parameter gathers CONGA 1 2, 4, 6, and 24 h) and MIME ΔG relationships were respectively assessed in 6/27 and 4/27 studies, mainly with glucose overall exposure markers. Five relationships between CONGAs and glucose overall exposure markers were studied including four correlation analyses (one non-significant, one negative, and two positive) and one non-significant linear regression analysis on 220 T2D patients. Five relationships between MIME ΔG and glucose overall exposure markers were studied including four correlation analyses (two negative and two positive) and one positive mixed model analysis on 162 T2D patients. We noticed the lack of evaluated relationships for both, CONGAs and MIME ΔG, and the other T2D or complication related markers.

##### Other parameters

**Figure 2:**
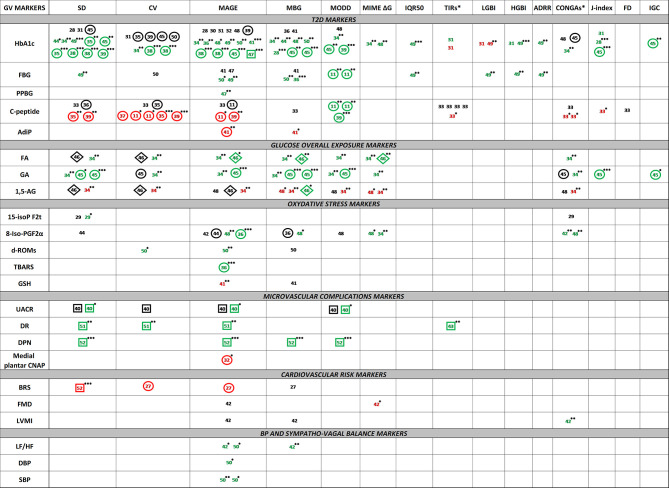
Visual representation of the relationships between GV parameters and T2D or metabolic disorder-related markersNumbers represent the study reference in which the relationship was tested. Green color represents the positive relationships. Red color represents the negative relationships. Black color represents the non-significance of relationships. p-values are indicated with superscripted stars (*: ≤ 0.05, **: ≤ 0.01, ***: ≤ 0.001). The absence of superscripted stars for colored numbers indicates the absence of p-value data. Naked numbers indicate correlation analysis, encircled numbers indicate linear regression analysis, numbers framed by a squared indicate logistic regression analysis, numbers framed by a diamond indicate mixed model analysis. BP, blood pressure; FBG, fasting blood glucose; PPBG, postprandial blood glucose; AdiP, adiponectin; FA, fructosamine; GA, glycated albumin; 1,5-AG, 1,5-anhydroglucitol; 15-isoP F2t, 15 F2t isoprostane; 8-Iso-PGF2α, 8-iso-Prostaglandin F2α; d-ROMs, derivatives of reactive oxygen metabolites; TBARS, thiobarbituric acid reactive substances; GSH, glutathione; UACR, urinary albumin/creatinine ratio; DR, diabetic retinopathy; DPN, diabetic peripheral neuropathy; medial plantar CNAP, compound nerve action potential; BRS, baroreflex sensitivity; FMD, flow-mediated dilation; LVMI, left ventricular mass index; DBP, diastolic blood pressure; SBP, systolic blood pressure; LF/HF, low frequency/high frequency ratio; SD, standard deviation; CV, coefficient of variation; MAGE, mean amplitude of glucose excursions; MODD, mean of daily difference; MIME, mean indices of meal excursions; IQR50, interquartile 50; TIR, time in range; LBGI, low blood glucose index; HBGI, high blood glucose index; ADRR, average of daily risk ratio; Min Gly, minimum of glycemia; Max Gly, maximum of glycemia; CONGA, continuous overall of net glycemic action; LAGE, largest amplitude of glycemic excursion; MBG, mean blood glucose; FD, fractal dimension. CONGA* gathers CONGA 1, 2, 4, 6, 24 (report to the articles for the evaluated intervals). TIR* gathers TIR <54 mg/dl, TIR <70 mg/dl, TIR >126 mg/dl, TIR [70–180 mg/dl] and TIR >180 mg/dl (report to the articles for the evaluated intervals).

We could not find any evaluated relationships of MIME ΔT or MIME BR in our analyzed studies. For all other GV parameters, such as TIR, LGBI, HGBI, ADRR, J-index, FD, or IGC, only few studies were focusing on potential relationships with some T2D or complication related markers. **Figure 2** synthesizes studies which have assessed at least one GV parameter and its relationship with at least one T2D or complication-related marker. The supplemental table (**Table S2**) gives more information (title, population, study duration…) about each study indicated in **Figure 2**. Full characteristics of included studies are presented in supplemental data (**Table S1**).

GV parameters and T2D markers (including glycemic control markers and T2D complication related markers) showed numerous relationships. SD, CV, MBG, and MAGE were the GV parameters the most studied, showing strong relationships, especially with HbA1c. The following step is to study these GV parameters as outcomes in an interventional study in T2D management.

#### Part 2: GV as an Outcome for Improving T2D management

Article screening is presented in the flowchart (**Figure 1**). The initial search identified a total of 243 records. After scanning titles and abstracts, 62 were accepted for further screening, and full-text of these studies was reviewed. Of these, 37 studies were identified as meeting the inclusion criteria. In addition, one article was identified through other sources during the process. In total, 38 articles were included in the first analysis. Common reasons for exclusion were: inclusion criteria not fulfilled, languages other than English or French, duplicates and results or T2D data unavailability.

**Figure 3:**
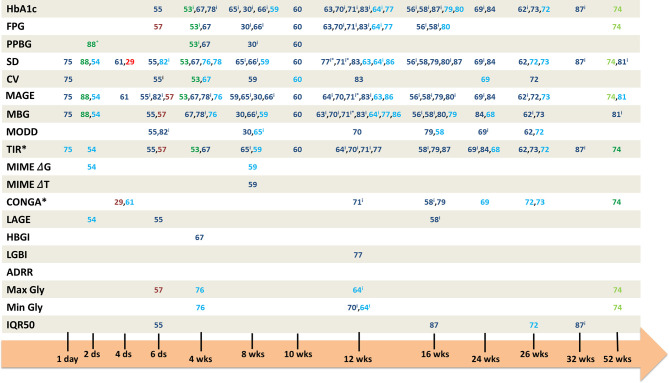
Timeline of the interventional studies evaluating GV parameters as outcomes. Numbers represent the interventional study reference in which the associated GV parameter was taken as an outcome. In blue numbers, the pharmacological studies, in green numbers, the nutritional studies, and in red numbers, the studies on physical activity. The dark colors represent the significant changes, and the light ones the non-significant changes. iintra-group comparison. ds, days; wks, weeks; FBG, fasting blood glucose; PPBG, postprandial blood glucose; SD, standard deviation; CV, coefficient of variation; MAGE, mean amplitude of glucose excursions; MODD, mean of daily difference; MIME, mean indices of meal excursions; IQR50, interquartile 50; TIR, time in range; LBGI, low blood glucose index; HBGI, high blood glucose index; ADRR, average of daily risk ratio; Min Gly, minimum of glycemia; Max Gly, maximum of glycemia; CONGA, continuous overall of net glycemic action; LAGE, largest amplitude of glycemic excursion; MBG, mean blood glucose; FD, fractal dimension. TIR*: report to the articles for glycemic ranges, CONGA*: report to the articles for the evaluated intervals. 71*: data for log(MBG), log(MAGE) and log(SD), 77*: only for SDdaytime, 88*: only for breakfast and lunch.

The characteristics of studies included in this analysis are presented in supplemental data (**Table S3**). Among the 38 interventional studies, 33 were based on pharmacological interventions, three on nutritional interventions and two on physical activity interventions. GV parameters were recorded with classical CGMS devices on 37 studies and for one study, a flash glucose monitoring system (FGMS) was used, being one of the latest and improved versions of CGMS. Among the 38 studies, 10 studies were conducted on T2D using insulin as basal treatment (among others), nine studies on T2D patients under metformin only, seven studies with T2D without any oral antidiabetic (OAD) medication, and the remaining12 studies were dealing with T2D patients under other OADs, in mono or combination therapy, or with non-homogeneous treatments between them (but without insulin). **Figure 3** represents the timeline of the studies including GV parameters as one of their outcomes. The supplemental table (**Table S4**) gives some information (title, population, study duration…) about each study indicated in **Figure 3**. Full characteristics of included studies are presented in supplemental data (**Table S3**).

##### Pharmacological Studies

Concerning the intervention duration, 13 studies were conducted in less than 3 months; for 15 studies, interventions were conducted during 3 to 6 months and five studies were conducted during 6 months to 1 year. The range of diabetes duration was quite spread, going from the newly diagnosed patients to patients with 18.6 years of diabetes. The basal HbA1c level in these pharmacological studies was between 6.5 and 10.6%, with a majority of studies dealing with patients having an HbA1c around 8.5%.

##### Nutritional and Physical Activity-Based Studies

The intervention durations of those studies were shorter than the ones observed for pharmacological studies (between 2 days and 4 weeks). One nutritional study lasted 52 weeks; however, on the eight parameters evaluated, only two appeared to be significantly improved after the intervention. We also noticed a difference with the pharmacological studies regarding the diabetes duration with a duration ≤9 years for the five N-AP studies, although patients were under a basal treatment in three studies. The basal HbA1c, between 5.1 and 7.3%, was also lower than the one in pharmacological studies.

##### GV parameters

###### Pharmacological Studies

The most studied GV parameters are SD (29 studies), MAGE (29 studies), and MBG (26 studies). TIR is also one of the most studied parameters with 20 studies dealing with it. MODD, CV, and CONGA followed with ten, eight, and seven studies respectively. All other GV parameters such as MIME, LBGI, HBGI, LAGE, or IQR50 are studied in only few studies. ADRR does not appear as an outcome in the selected studies.

For SD, on 29 pharmacological studies, 20 showed a significant decrease after intervention or between treatments. Concerning MAGE, 23/29 studies found a significant decrease for this parameter and for MBG, 18/26 studies appeared to be significant for MBG decrease. Regarding the TIR, all intervals taken together, 16/20 studies showed significant changes after intervention or between treatments. Three of the ten studies focusing on MODD did not show any significant changes, 3/8 for SD, and 4/7 for CONGA (all intervals taken together).

##### N-AP Studies

In our analysis, we identified only five studies dealing with N-AP interventions with GV parameters as outcomes (three nutritional studies and two based on physical activities). For these studies, SD and MAGE are the most represented GV parameters with four studies assessing them as outcomes. TIR comes after with three studies, followed by MBG and CONGA with two studies. Like the pharmacological studies, IQR50, ADRR, LBGI, HBGI, LAGE, and MIME were not taken as outcomes in N-AP studies. However, unlike pharmacological studies, MODD is not part of the outcomes in the N-AP studies, and CV has only been identified once as outcome.

For SD, on four N-AP studies, 2/4 showed a significant decrease after intervention or between treatments. Concerning the MAGE, 3/4 studies found a significant decrease for this parameter and for the TIR, all intervals taken together, all the identified studies showed significant changes after intervention or between treatments. We found the same pattern for MBG and CONGA.

##### GV and glycemic control

###### Pharmacological Studies

Of 21 studies assessing both MAGE and HbA1c, 15 were associated with both significant improvement in MAGE and HbA1c. Despite the fact that no significant changes in HbA1c occurred in five interventional studies, significant decreases in MAGE were highlighted in the same five studies. The same pattern was found for changes in both MAGE and FPG with 12 studies assessing both parameters. Among these 12 studies, eight studies showed an improvement in both MAGE and FPG. Two studies showed no changes in FBG but did show significant decrease in MAGE. Concerning MAGE and PPBG, three studies were dealing with both parameters, all showing significant decrease for both parameters. The same studies’ repartition pattern occurred for results concerning SD, MBG or TIR, and glycemic control parameters (HbA1c, FBG, and PPBG).

##### N-AP Studies

Only two studies assessed both MAGE and HbA1c. One study did not show any significant changes for both parameters, and the other one was associated with significant improvement in both MAGE and HbA1c. However, this previous study was conducted for 4 weeks which may be too short to evaluate HbA1c modification. Significant improvements in both MAGE and FPG were found in 2/3 studies; the last one showing no significant changes for both parameters. We found significant improvements for both MAGE and PPBG in the two studies dealing with these parameters. The same studies’ repartition pattern occurred for results concerning SD and glycemic control parameters (HbA1c, FBG, and PPBG). Concerning both TIR and HbA1c evaluated in two studies, taking off the study conducted in 4 weeks, one study showed a significant improvement in TIR but not in HbA1c. Significant improvements were found for both TIR and FBG for 2/3 studies, the third study showing only improvement in TIR but not in FPG. The only study dealing with both TIR and PPBG showed significant improvement in both parameters. Concerning CONGA, one study assessed this parameter with both HbA1c and FPG and showed a significant improvement in CONGA only. MBG was found to be significantly improved with PPBG in the only study dealing with both parameters.

## Discussion

Concerning the relationships of GV parameters with T2D diagnosis parameters and T2D-related complications, among GV parameters, SD, CV, MBG, and MAGE appear as the most studied ones, showing strong relationships, particularly with HbA1c. Regarding the investigation on the use and relevance of GV as a relevant outcome in interventional studies, in the pharmacological ones, SD, MAGE, MBG, and TIR are the GV parameters used as outcomes in the majority of the studies, showing significant improvement alone but also in parallel with glycemic control parameters (HbA1c, FBG, and PPBG). In the N-AP interventional studies, SD, MAGE, and TIR stand out as outcomes, showing also significant improvement. Most of the identified studies are dealing with pharmacological intervention and until day only few are focusing on lifestyle intervention (nutrition and physical activity).

### Relationships Between GV Parameters and T2D Related Markers

In the first part of our analysis, the large majority of the studies showed significant relationships between GV parameters and T2D or metabolic disorder-related markers. Concerning CV and SD, whether studies evaluate correlation analyses or linear regression analyses with adjustment on some parameters, the majority of the studies showed positive relationships with T2D or metabolic disorder-related markers. For MAGE, studies mainly focus on correlation analyses, but the significance and the direction of tested relationships are maintained with regression analyses. However, there are discrepancies, and some studies failed to show such associations which can be explain partly by the basal treatment or comorbidities of patients included in such studies.

Regarding the treatment, in the study of Craciun et al. (33), 22.6% of the patients are under insulin in mono or combination therapy, and such diversity of treatment could have an effect on the non-significance of association for the most known GV parameters. The same study failed to demonstrate associations for some GV markers; yet, this study focused on the C-peptide linear regressions and effect on the glycemic variability parameters in patients with type 2 diabetes. Endogenous insulin secretion, which can be evaluated by C-peptide levels (89), varies widely among T2D patients, which is reinforced here by the diversity of the study on T2D patients’ basal treatment. In addition, some computation using fasting C-peptide is not valid in patients on insulin therapy (89). In the study of Jin et al. (35), which looked at two groups of patients with different therapies, associations between C-peptide and SD or CV appear to be significant in T2D patients under insulin therapy, whereas same associations are lost when looking at T2D patients without insulin therapy. The same conclusions about insulin treatment and C-peptide association with CV or SD are provided by Christensen et al. (37), Huang et al. (39), and Ohara et al. (90).

Regarding comorbidities, the study conducted by Jin et al. (38) also reports the non-significance of logistic regressions for T2D patients having a UACR between 30 and 299 mg/g, revealing microalbumineria, whereas logistic regressions are significant for those having albuminuria with UACR ≥300 mg/g which could indicate that the presence and/or the severity of kidney disease may affect relations and clinical relevance of some GV parameters.

Concerning the potential mechanisms linking GV and diabetic complications, it has been demonstrated that chronic hyperglycemia, defined by both fasting and postprandial hyperglycemia, is involved in the development of both micro and macro vascular complications associated with diabetes (8, 103). Chronic hyperglycemia status leads to oxidative stress, following an imbalance between free radicals and antioxidant species production in favor of free radicals or reactive oxygen species production (104, 105). This oxidative stress is involved in the pathophysiology of diabetes complications. Glycation reactions may also be promoted and advanced glycation products may be formed and involved in the development of oxidative stress and pro-inflammatory status (105), leading to an abnormal production of pro-inflammatory cytokines and an activation of the inflammatory signaling pathways (106).

### Relevance of GV Parameters in Continuous Glucose Monitoring Regarding More Traditional T2D Markers

An interesting point to notice in the second part of our analysis is the presence, in some studies, of significant changes in GV parameters, whereas glycemic control parameters did not reach significance. Indeed, in addition to MAGE, without changes in HbA1c, 5/5 studies showed improvement in TIR, 3/6 in SD, and 3/7 in MBG. There are no particularly common features in intervention, basal treatment, or diabetes duration between all these studies. The only differences that we found concerning some baseline parameters such as diabetes duration, study duration, or HbA1c levels, depended on the type of study (pharmacological *vs* N-AP interventions). The majority of studies dealing with HbA1c as outcome showed significant improvements lasted 12 weeks or more. GV parameters could be modulated in a shorter term and appear to be related to HbA1c. In our analysis, for example, 16/23 studies showed improvement in both HbA1c and MAGE, 14/14 in both HbA1c and TIR, 14/18 in both HbA1c and SD, and 15/15 in both HbA1c and MBG. Using such GV parameters as intermediate markers to assess glycemic control when HbA1c is not available due to the study duration could be an interesting alternative to evaluate drug or food impact on T2D patients, providing complementary information to HbA1c. For the majority of studies lasting less than 12 weeks, evaluated GV parameters showed significant changes after intervention which reinforces the previous idea and shows that CGMS could be a sensitive tool to assess GV and glycemic control in shorter studies when HbA1c cannot be assessed.

### Assessment and Relevance of Different GV Parameters as Outcomes for Intervention Targeting Glucose Control

In interventional studies, most GV parameters evaluated as outcomes appear to be MAGE, SD, MBG, and TIR with significant improvements after intervention in patients with T2D. However, some GV parameters are less studied but could have a relevant interest targeting more specific periods (postprandial period for example) and bringing more specific information such as the time to reach the glycemic peak.

In interventional studies, TIR is quite well identified with significant changes and could be seen as a valuable GV parameter for routine assessment; it is able to give information on the time spent in different glycemic targets. It appears to be relevant for targeting interventions aiming at controlling glycemic response and can be easily measured and evaluated in clinical practice. Before 2017, TIR glycemic ranges were not standardized, as can be seen in the identified studies for both analyses, which made comparisons between studies hard to assess. In 2017, the *International Consensus in Time In Range* has standardized glycemic ranges, and TIR was proposed as relevant outcome for clinical trials in addition to fasting glycemia or HbA1c (91). Besides, TIR is easily computable, and thanks to visual representations such as pie chart or histogram, it can be easily understood by the patient and used as a relevant support as well for educational therapy.

MIME ΔG appears to be a GV parameter more evaluated in studies assessing marker relationships than in interventional studies. This parameter, MIME ΔG, focuses on the glycemic peak after a meal and its assessment could be of interest in interventional studies for T2D because it gives comprehensive information related directly to the post-meal glycemic excursions. This metric gathers also the ΔT, which is the time to reach the peak, and RB which gives information about the return to basal value. Both are not often evaluated in studies but may deserve more attention because they are the only ones providing information about the postprandial kinetics; it could also be interesting especially for nutritional interventions focusing on carbohydrate quality which could impact the glycemic response profiles in T2D.

Some GV parameters are less represented in both review parts. For example, J-index, fractal dimension (FD), and index of glycemic control (IGC) were evaluated in the first analysis only and not at all in the interventional studies. J-index perpetuates the inclusion of SD into the measurement of glycemic variability and represents the measure of both the mean level and variability of glycemia (22, 92). It was first designed for intermittent BG determinations then adapted to continuous monitoring data; its formula depends on SD and MBG which could explain the lack of studies dealing with it. FD describes glucose variability of high frequency and small amplitude. This GV parameter was more widely studied in T1D than in T2D to assess hypoglycemia (93). The IGC represents the sum of the hyperglycemic index and hypoglycemic index (23), meaning that it takes into account LGBI and HGBI, two parameters more appropriate for T1D patients which may be more sensitive to large glucose fluctuations.

According to the ADA 2019 recommendations, GV, evaluated by a Continuous Glucose Monitoring System (CGMS) has an important role in assessing the effectiveness and safety of treatment in many patients with T1D. For T2D patients, limited data suggest that it could also be helpful, especially for those on intensive insulin regimens (12, 94). For those patients beyond real-time treatment adjustment, CGMS in real-time could be an interesting tool to assess glycemic profile and to help them in the management of the disease. In its masked version, CGMS appears as one of the most relevant tools to conduct clinical trials assessing glycemic profiles because patients cannot have access to their glycemic data in a real-time, thus limiting bias on results regarding, for example, the potential anxiety caused by hypo, hyperglycemia, or the food and medication auto-adjustment.

The list of GV parameters evaluated in this review is not exhaustive but gathers the most known assessed parameters today. Other GV metrics evaluating the quality of glycemic control based on “risk indices” could be used in gathering and combining GV parameters that assign larger penalty scores to glucose levels falling progressively further away from the target range (95). For example, the GRADE (Glycemic Risk Assessment Diabetes Equation), will assess the relationship between penalty score and glucose level based on average of numerical ratings from multiple categories of clinicians and healthcare professionals (96). Another one, the M_R_ value, focuses on the overall quality of glycemic control; adjustable parameter (R) affecting the relative importance of glucose values in the hypoglycemic and hyperglycemic ranges (97). Some of those GV metrics require complex mathematical computations and remain quite difficult to assess in clinical practice. Henceforth, defining GV parameters that are easily understandable and clinically appropriate appears to be necessary in the diabetes care (98) because even if there is no consensus on the GV metrics to use, there is one about the deleterious effect of such short-term glycemic fluctuations in T2D people, leading authors to propose them as additional treatment targets (26).

Of course, there is still a lack of studies on less known GV parameters, for which relationships with T2D markers are mainly evaluated with correlation analyses to reach a clear consensus concerning potential relationships with T2D glycemic control and metabolic disorder-related markers and concerning their relevance to assess glycemic control in interventional studies. Besides, it remains a difficult challenge to report direct relationships between GV parameters and global patient prognosis. To our knowledge, studies are dealing with several independent markers, and no direct data on patient prognosis are available. We therefore decided to identify intermediate related risk markers and related comorbidities to address the link between GV and patient evolution status.

Some GV parameters appear to be less relevant for T2D and more for T1D, which could explain the discrepancies in terms of relationships or effects in the studies. Because the most known GV parameters (SD, CV, MAGE) are widely studied, they appear to be the most relevant metrics to assess glycemic control in T2D patients, but some others as TIR or MIME could bring very useful additional information to go further in the appreciation of glycemic control and metabolic disorders related to T2D.

## Conclusion

Finally, GV appears as a key component of T2D dysglycemia and could be used in addition to classical markers of glycemic control such as HbA1c, FBG or PPBG. CGMS could be a sensitive tool to assess GV and glycemic control in shorter studies when HbA1c cannot be assessed. With technical progress, there are an increasing number of available tools to follow glycemia in a continuous way, and CGMS appears to be a relevant tool to assess it in clinical research. Pharmacological studies are in a predominant position but N-AP interventions, despite the small number of such studies dealing with both GV parameters and glycemic control ones, showed interesting results on GV parameters and would require more in-depth work. As we reported previously, it is very important to consider postprandial period in controlling glycemic response, especially in T2D. Indeed, humans spend more than ¾ of their lives in postprandial period, and although postprandial excursions have been widely studied, studies were mostly focusing on the area under the curve or the glycemic peak (99). Consequently, assessing GV parameters as outcomes in lifestyle studies could bring more integrative markers. Medical nutritional therapy holds a major position in T2D management with a recent increased interest in food quality and more particularly in carbohydrate quality, impacting postprandial glycemic response and cardiometabolic risks. In this perspective, CGMS could be used to evaluate effects of lifestyle interventions on glycemic control and postprandial periods.

## Author Contributions

A-EB, ML, SV, and J-AN contributed to the review design. A-EB contributed to article screening. S-LP, SV, and J-AN contributed to the verification of screened articles. A-EB and J-AN wrote the manuscript. All authors contributed to the article and approved the submitted version.

## Conflict of Interest

SV and A-EB are employees of Mondelez International.

The remaining authors declare that the research was conducted in the absence of any commercial or financial relationships that could be construed as a potential conflict of interest.

## Publisher’s Note

All claims expressed in this article are solely those of the authors and do not necessarily represent those of their affiliated organizations, or those of the publisher, the editors and the reviewers. Any product that may be evaluated in this article, or claim that may be made by its manufacturer, is not guaranteed or endorsed by the publisher.

## Supplementary Material

Supplementary Table 1 Characteristics of studies included in Part 1 analysis.

Supplementary Table 2 Summary of studies included in Part 1 analysis.

Supplementary Table 3 Characteristics of studies included in Part 2 analysis.

Supplementary Table 4 Summary of studies included in Part 2 analysis.

## Abbreviations

1; 5-AG, 1; 5-anhydroglucitol; 15-isoP F2t, 15 F2t isoprostane; 8-Iso-PGF2*α, *8-iso-prostaglandin F2α; ADA, American Diabetes Association; ADRR, average of daily risk ratio; BRS, baroreflex sensitivity; CGMS, continuous glucose monitoring system; CNAP, compound nerve action potential; CONGA, continuous overall net glycemic action; CV, coefficient of variation; DBP, diastolic blood pressure; DPN, diabetic peripheral neuropathy; DR, diabetic retinopathy; d-ROMs, derivatives of reactive oxygen metabolites; FA, fructosamine; FBG, fasting blood glucose; FD, fractal dimension; FGMS, flash glucose monitoring system; FMD, flow-mediated dilation; GA, glycated albumin; GRADE, glycemic risk assessment diabetes equation; GSH, glutathione; GV, glycemic variability; HBGI, high blood glucose index; IDF, International Diabetes Federation; IGC, index of glycemic control; IQR50, interquartile 50; LAGE, largest amplitude of glucose excursion; LBGI, low blood glucose index; LF/HF, low frequency/high frequency; LVMI, left ventricular mass index; MAGE, mean amplitude of glucose excursion; Max Gly, maximum of glycemia; MBG, mean blood glucose; MIME, mean indices of meal excursion; Min Gly, minimum of glycemia; MODD, mean of daily differences; N-AP, nutritional and physical activity; OAD, oral anti-diabetic; PPBG, postprandial blood glucose; RB, basal return; SBP, systolic blood pressure; SD, standard deviation; SMBG, self monitoring blood glucose; T1D, type 1 diabetes; T2D, type 2 diabetes; TBARS, thiobarbituric acid reactive substances; TIR, time in range; UACR, urinary albumin/creatinine ratio.
